# Inhibition of ITK Signaling Causes Amelioration in Sepsis-Associated Neuroinflammation and Depression-like State in Mice

**DOI:** 10.3390/ijms24098101

**Published:** 2023-04-30

**Authors:** Mohammad M. Algahtani, Samiyah Alshehri, Sana S. Alqarni, Sheikh F. Ahmad, Naif O. Al-Harbi, Saleh A. Alqarni, Ali S. Alfardan, Khalid E. Ibrahim, Sabry M. Attia, Ahmed Nadeem

**Affiliations:** 1 Department of Pharmacology and Toxicology, College of Pharmacy, King Saud University, Riyadh 11451, Saudi Arabiafashaikh@ksu.edu.sa (S.F.A.);; 2 Department of Medical Laboratory Science, College of Applied Medical Sciences, King Saud University, Riyadh 11451, Saudi Arabia; 3 Department of Zoology, College of Science, King Saud University, Riyadh 11451, Saudi Arabia

**Keywords:** sepsis, ITK, neuroinflammation, IL-17A, depression

## Abstract

Sepsis affects millions of people worldwide and is associated with multiorgan dysfunction that is a major cause of increased morbidity and mortality. Sepsis is associated with several morbidities, such as lung, liver, and central nervous system (CNS) dysfunction. Sepsis-associated CNS dysfunction usually leads to several mental problems including depression. IL-17A is one of the crucial cytokines that is expressed and secreted by Th17 cells. Th17 cells are reported to be involved in the pathogenesis of depression and anxiety in humans and animals. One of the protein tyrosine kinases that plays a key role in controlling the development/differentiation of Th17 cells is ITK. However, the role of ITK in sepsis-associated neuroinflammation and depression-like symptoms in mice has not been investigated earlier. Therefore, this study investigated the efficacy of the ITK inhibitor, BMS 509744, in sepsis-linked neuroinflammation (ITK, IL-17A, NF*k*B, iNOS, MPO, lipid peroxides, IL-6, MCP-1, IL-17A) and a battery of depression-like behavioral tests, such as sucrose preference, tail suspension, and the marble burying test. Further, the effect of the ITK inhibitor on anti-inflammatory signaling (Foxp3, IL-10, Nrf2, HO-1, SOD-2) was assessed in the CNS. Our data show that sepsis causes increased ITK protein expression, IL-17A signaling, and neuroinflammatory mediators in the CNS that are associated with a depression-like state in mice. ITK inhibitor-treated mice with sepsis show attenuated IL-17A signaling, which is associated with the upregulation of IL-10/Nrf2 signaling and the amelioration of depression-like symptoms in mice. Our data show, for the first time, that the ITK inhibition strategy may counteract sepsis-mediated depression through a reduction in IL-17A signaling in the CNS.

## 1. Introduction

Sepsis is associated with multiorgan dysfunction that may lead to increased morbidity and mortality. The prevalence of sepsis is reported to be 300–500 cases/100,000 people in the world. It is one of the leading causes of death in intensive care units with as many as 11 million sepsis-associated deaths worldwide per year [1,2,3,4]. Sepsis is associated with several morbidities, such as kidney, liver, cardiac, and central nervous system (CNS) dysfunction. Sepsis-associated encephalopathy causes a variety of mental problems, such as depression, delirium, and dementia. As many as 70% of critically ill patients are associated with sepsis-associated encephalopathy, which is a major cause of increased mortality and hospital costs for survivors especially in elderly people [4,5,6,7]. Therefore, there is a need for early intervention that may block the inflammatory pathways leading to neuroinflammation.

ITK is a member of the Tec kinase family and plays a quintessential function in T lymphocyte development and differentiation, i.e., CD4+ T cells, CD8 + T cells, NKT cells, and γδ T cells. ITK becomes activated through cell surface receptors on T cells, i.e., TCR and causes the activation of downstream signaling leading to the modulation of transcriptional machinery [8,9]. Transcriptional factors such as NFATc1 and STAT3 are regulated by ITK in naïve CD4+ T cells (Th0), which convert these cells into Th17 cells [8,10,11]. ITK signaling is also known to negatively regulate the development of Foxp3 expressing regulatory T cells (Treg) [12,13]. Hence, ITK has been shown to be overactivated in a variety of disease states, such as autoimmune neuroinflammatory disorders, acute lung/kidney injury, and psoriatic inflammation in animals [14,15,16]. Based on these observations, several ITK inhibitors have been shown to cause the downregulation of inflammatory pathways in immune-mediated autoimmune/inflammatory diseases, such as psoriasis, asthma, multiple sclerosis, and ulcerative colitis in both humans and animals [10,17,18]. However, sepsis-associated neuroinflammation remains uninvestigated with respect to ITK signaling.

It has been reported in the past that there is bidirectional communication between immune cells and CNS. T cells may contribute towards neuroinflammation via the expression of pro-inflammatory mediators. These inflammatory signals may include costimulatory signals or pro-inflammatory cytokines [7,19,20]. Other immune cells, such as neutrophils, which express iNOS and MPO, may also promote neuroinflammation through the induction of BBB dysfunction [21,22,23]. This may allow a greater infiltration and accumulation of other immune cells such as monocytes/T cells/B cells from systemic circulation into the CNS during sepsis. Infiltrated immune cells further amplify neuroinflammation by activating microglial cells, which release different pro-inflammatory chemokines, cytokines, and oxidants [24,25]. However, how ITK signaling affects oxidative inflammation in the CNS remains unexplored with respect to sepsis.

Therefore, this study attempted to explore the role of ITK signaling in causing systemic inflammation and neuroinflammation associated with sepsis. Further, the ITK inhibition approach was utilized to affirm whether ITK signaling was associated with depression-like effects in sepsis survivor mice. Our data reveal increased *p*-ITK protein levels in circulating CD4+ T cells and ITK protein levels in the cerebral cortex, which are associated with sepsis-mediated neuroinflammation and depression-like symptoms. The ITK inhibitor, BMS 509744, causes significant attenuation in sepsis-associated neuroinflammation and depression-like behavior through the downregulation of Th17-related mediators and oxidative stress in peripheral immune cells and the CNS.

## 2. Results

### 2.1. Sepsis Causes Upregulation of ITK in the Periphery and CNS 

**Figure 1:**
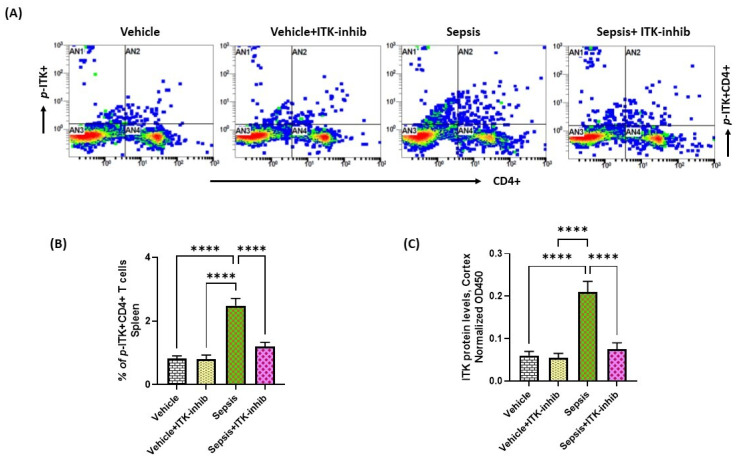
Elevation of ITK signaling in the periphery and CNS of sepsis survivor mice. (**A**) Representative flow plot for *p-ITK+* CD4+ T cells’ immunostaining; (**B**) % of *p-ITK+* CD4+ T cells; and (**C**) ITK protein levels in the cerebral cortex. Values are depicted as the mean ± SEM, *n* = 6–8/group. **** *p* < 0.0001.

Sepsis is known to be associated with systemic inflammation and neuroinflammation in which ITK signaling may be involved; therefore, the modulation of ITK signaling in these phenomena was examined in this study. Our study showed an increased activation of ITK in CD4+ T cells as reflected by an increased % of *p-ITK+* CD4+ T cells in the periphery of sepsis survivor mice (Figure 1A,B). Further, the ITK protein levels were also elevated in the cerebral cortex of sepsis survivor mice (Figure 1C). Treatment with the ITK inhibitor caused a downregulation in the *p-ITK+* CD4+ T cells and ITK levels in the CNS of sepsis survivor mice (Figure 1). These data highlight that ITK signaling becomes activated in the CD4+ T cells and CNS of sepsis survivor mice.

### 2.2. Sepsis-Induced Elevation in Th17 Cell-Related Signaling Is Attenuated by ITK Inhibition

**Figure 2:**
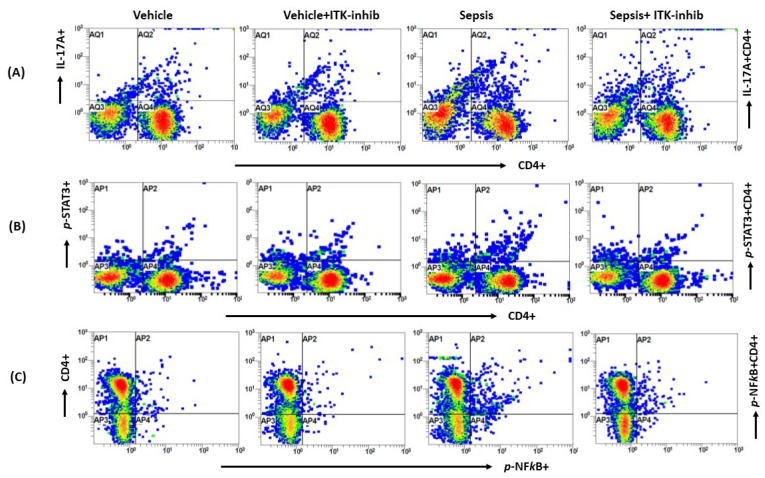

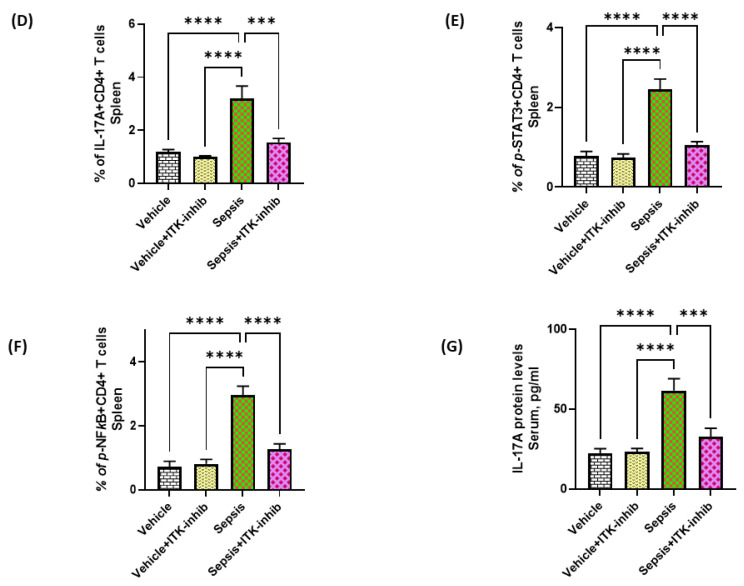
The ITK inhibitor downregulated Th17-related signaling in the periphery of sepsis survivor mice. (**A**) Representative flow plot for IL-17A+ CD4+ T cells’ immunostaining; (**B**) Representative flow plot for *p-STAT3+* CD4+ T cells’ immunostaining; (**C**) Representative flow plot for *p-NFkB+* CD4+ T cells; (**D**) % of IL-17A+ CD4+ T cells; (**E**) % of *p-STAT3+* CD4+ T cells; (**F**) % of *p-NFkB+* CD4+ T cells; and (**G**) IL-17A protein levels in serum. The values are depicted as the mean ± SEM, *n* = 6/group. *** *p* < 0.001; **** *p* < 0.0001.

Next, we wanted to explore whether Th17-related immune responses are controlled by ITK signaling in periphery and the CNS, and whether the BMS compound is able to attenuate them. Our data show that IL-17A, *p*-STAT3, and *p*-NF*k*B are elevated in CD4+ T cells in the periphery as depicted by the % of IL-17A+ CD4+ T cells, *p-STAT3+* CD4+ T cells, and *p-NFkB+* CD4+ T cells (Figure 2). Sepsis-induced elevation in the Th17-related parameters were attenuated by the ITK inhibitor in the periphery (Figure 2). 

### 2.3. Sepsis-Induced Elevation in IL-17A Signaling Is Attenuated by ITK Inhibition

**Figure 3:**
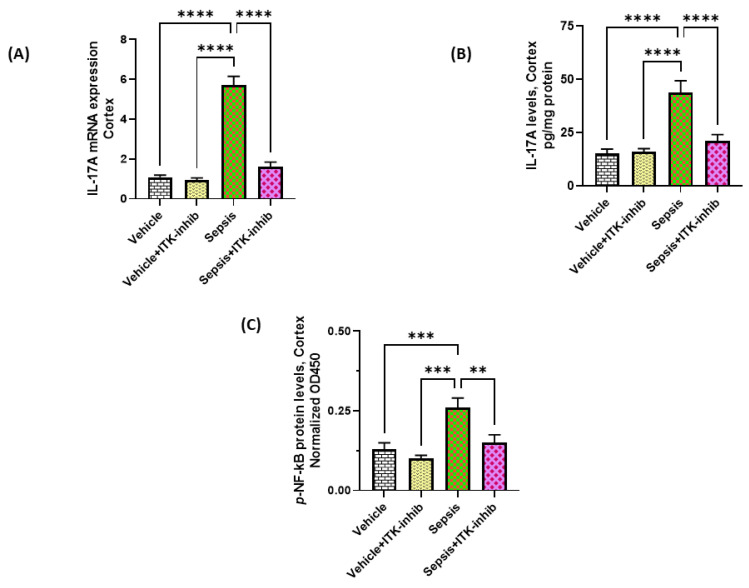
ITK inhibitor caused a reduction in IL-17A-related signaling in the CNS of sepsis survivor mice. (**A**) IL-17A mRNA expression; (**B**) IL-17A protein levels; and (**C**) *p*-NF*k*B protein levels. The values are depicted as the mean ± SEM, *n* = 6–8/group. ** *p* < 0.01; *** *p* < 0.001; **** *p* < 0.0001.

Further, IL-17A-mediated signaling in the cerebral cortex was also explored. Our data show elevated IL-17A levels in the cerebral cortex, which were associated with elevated *p*- NF*k*B protein levels (Figure 3A–C). Sepsis-induced IL-17A signaling was greatly reduced by the ITK inhibitor (Figure 3). *p*-STAT3 protein levels in the cerebral cortex were also significantly increased in the sepsis group (0.183 ± 0.02, mean ± SEM, *n* = 8) as compared to the vehicle (0.097 ± 0.01, mean ± SEM, *n* = 6; *p* < 0.01) and the vehicle + ITK-inhib (0.103 ± 0.012, mean ± SEM, *n* = 6; *p* < 0.01) groups; however, the sepsis + ITK-inhib group (0.155 ± 0.015, mean ± SEM, *n* = 8; *p* > 0.05) did not have any significant difference in the *p*-STAT3 levels as compared to the sepsis group (0.183 ± 0.02, mean ± SEM, *n* = 8). These data show that ITK activation is involved in the elevation of IL-17A levels both in the periphery and the CNS, which can be controlled by ITK inhibition.

### 2.4. Sepsis-Induced Oxidative Neuroinflammation Is Attenuated by ITK Inhibition

**Figure 4:**
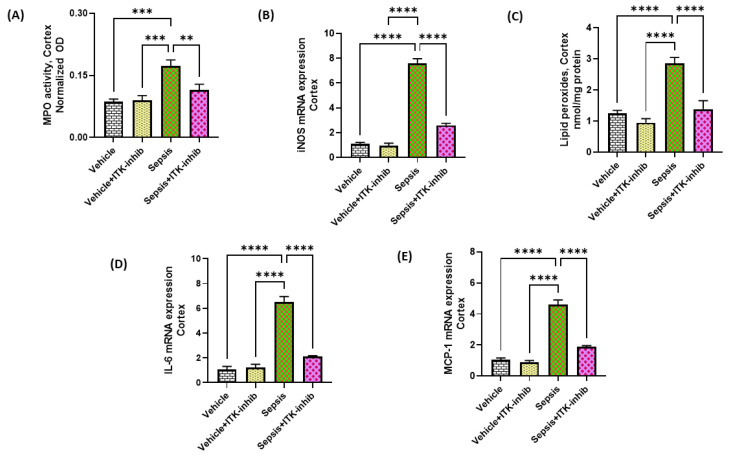
ITK inhibitor caused a reduction in oxidative and inflammatory mediators in the CNS of sepsis survivor mice. (**A**) MPO activity; (**B**) iNOS mRNA expression; (**C**) Lipid peroxides; (**D**) IL-6 mRNA expression; and (**E**) MCP-1 mRNA expression. The values are depicted as the mean ± SEM, *n* = 6–8/group. ** *p* < 0.01; *** *p* < 0.001; **** *p* < 0.0001.

Oxidative inflammation is a major contributor in neuroinflammatory pathways associated with sepsis; therefore, we next explored whether ITK inhibition would lead to a modulation of the oxidative neuroinflammation. Our data show that the MPO activity, iNOS, and lipid peroxides were elevated in the cerebral cortex of mice with sepsis (Figure 4A–C). Treatment with BMS 509744 caused a significant downregulation of the oxidative markers in the cerebral cortex of sepsis survivor mice (Figure 4A–C). Further, it was evaluated whether the ITK inhibitor also caused the downregulation of neuroinflammatory mediators in sepsis survivor mice. Our data reveal that IL-6 and MCP-1 mRNA were elevated in the cerebral cortex of sepsis survivor mice (Figure 4D,E). The sepsis-associated elevation in neuroinflammatory mediators, such as IL-6 and MCP-1, were greatly reduced in the CNS by the ITK inhibitor (Figure 4D,E). These data show that the ITK inhibition approach has the potential to attenuate sepsis-induced oxidative and neuroinflammatory mediators. 

### 2.5. Treg Cell-Related Signaling Is Further Elevated by ITK Inhibition in Sepsis Survivor Mice

**Figure 5:**
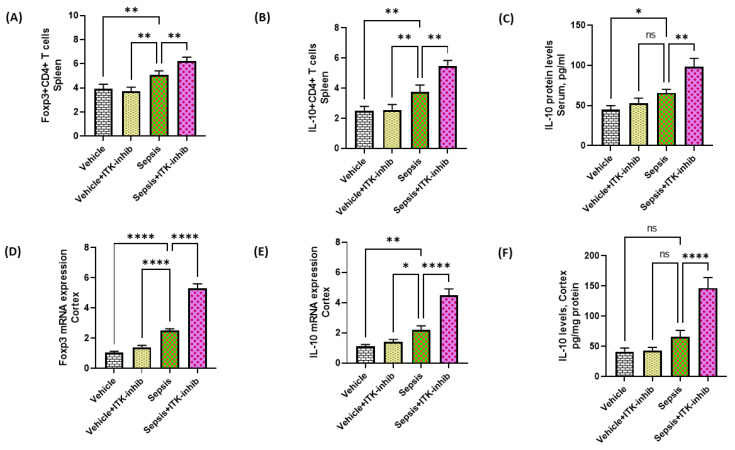
The ITK inhibitor caused an elevation in Treg-related signaling in the periphery and CNS of sepsis survivor mice. (**A**) % of Foxp3 + CD4+ T cells; (**B**) % of IL-10+ CD4+ T cells; (**C**) IL-10 protein levels in serum; (**D**) Foxp3 mRNA expression; (**E**) IL-10 mRNA expression; and (**F**) IL-10 protein levels in the cortex. The values are depicted as the mean ± SEM, *n* = 6–8/group. * *p* < 0.05; ** *p* < 0.01; **** *p* < 0.0001; ns = not significant.

ITK is also known to regulate the development of Treg cells. Therefore, the effect of the ITK inhibitor was investigated on Treg-related parameters in the periphery and CNS. Our data show that IL-10 and Foxp3 are elevated in CD4+ T cells in the periphery of sepsis survivor mice as depicted by the % of IL-10+ CD4+ T cells and Foxp3+ CD4+ T cells (Figure 5A–C). Treg cells were further elevated by the ITK inhibitor in sepsis survivor mice (Figure 5A–C). Further, IL-10 and Foxp3 mRNA levels were also elevated in the cerebral cortex of sepsis survivor mice by the ITK inhibitor (Figure 5D–F). These data show that ITK inhibition upregulates Treg-related signaling in the periphery and CNS, which may attenuate sepsis-induced neuroinflammation.

### 2.6. Sepsis-Mediated Decrease in Antioxidant Transcription Factor Nrf2 Is Restored by ITK Inhibition in the CNS

**Figure 6:**
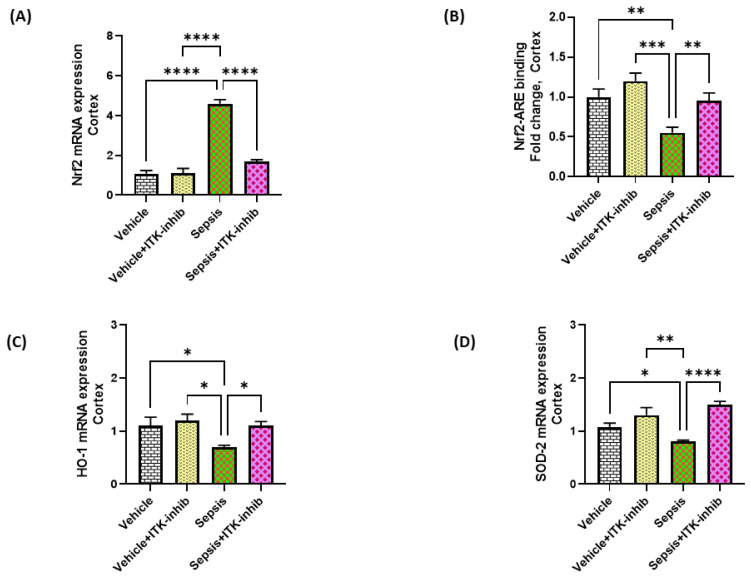
The ITK inhibitor caused a restoration of the Nrf2 signaling in the CNS of sepsis survivor mice. (**A**) Nrf2 mRNA expression; (**B**) Nrf2–ARE binding in the cortex; (**C**) HO-1 mRNA expression; and (**D**) SOD2 mRNA expression. The values are depicted as the mean ± SEM, *n* = 6–8/group. * *p* < 0.05; ** *p* < 0.01; *** *p* < 0.001; **** *p* < 0.0001.

Further, the effect of BMS 509744 was assessed on the Nrf2 antioxidant system. Nrf2/HO-1 signaling was dysregulated in the cerebral cortex as depicted by the decreased nuclear translocation of Nrf2 in mice with sepsis, although the Nrf2 mRNA levels were increased in the same group (Figure 6A,B). Consequently, Nrf2-induced antioxidant enzymes were dysregulated in the cortex as depicted by the decreased HO-1 and SOD-2 mRNA levels in the sepsis survivor mice (Figure 6C,D). Sepsis-induced derangements in Nrf2 signaling were corrected by the ITK inhibitor as reflected by the restoration of Nrf2 nuclear translocation in the cerebral cortex (Figure 6).

### 2.7. Sepsis-Induced Depression-like Behavior Is Ameliorated by ITK Inhibition

**Figure 7:**
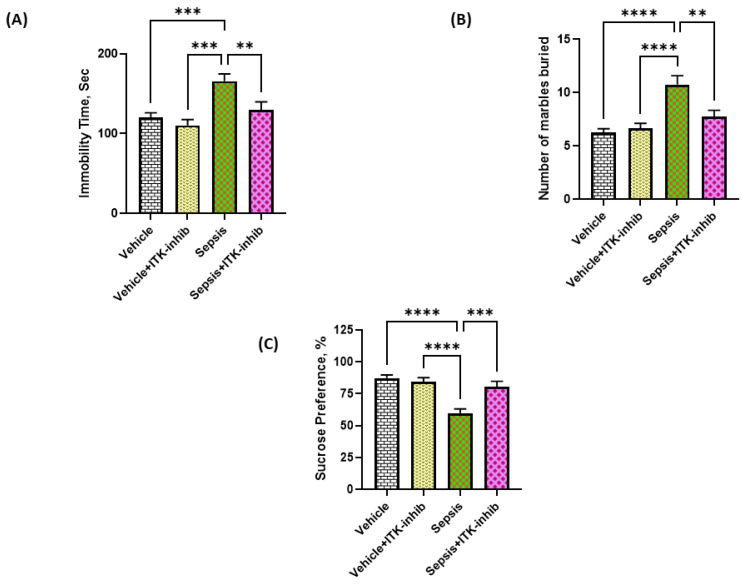
The ITK inhibitor caused an amelioration of the sepsis-induced depression-like state in mice. (**A**) Immobility time in TST; (**B**) Marble burying behavior; and (**C**) Sucrose preference test. The values are depicted as the mean ± SEM, *n* = 6–8/group. ** *p* < 0.01; *** *p* < 0.001; **** *p* < 0.0001.

Sepsis is known to induce depression-like behavior after the recovery period. Therefore, we finally investigated whether ITK inhibition has the potential to modulate depression-like behavior in sepsis survivor mice. Our data display that mice with sepsis survival had depression-like behavior. Mice that recovered from sepsis had an increased immobility time in the tail suspension test, an increased marble burying character, and a decreased sucrose preference indicative of depression-like behavior (Figure 7A–C). BMS 509744 caused a reversal of sepsis-induced depression-like behavior as displayed by a decrease in immobility time and a decrease in the number of marbles buried (Figure 7A,B). Further, BMS 509744 caused a reversal of the sepsis-induced reduction in sucrose preference (Figure 7C). These data show that the sepsis-induced depression-like state is ameliorated by the ITK inhibition strategy.

## 3. Discussion

Immune dysfunction in sepsis is characterized by dysregulated immune responses, which are orchestrated by both arms of the immune system, i.e., the innate (neutrophils/DCs) and adaptive (T cells/B cells) arms. The innate immune system is initially activated by different microbial agents in sepsis, which causes the release of a variety of mediators that include oxidants, chemokines, prostaglandins, and cytokines [7,26,27,28]. This systemic inflammatory soup causes an activation of the adaptive immune system as well as other structural cells ultimately leading to a hyperinflammatory state that causes neuroinflammation and behavioral derangements [8,29,30]. Our study shows that ITK plays an important role in sepsis-associated neuroinflammation through the modification of Th17-related signaling in the periphery and CNS.

The Tec kinase family consists of several members that include TEC, BTK, TXK, BMX, and ITK. ITK is favorably expressed in T cells where ITK expression is the highest [14,15,16]. Apart from T cells, ITK is also expressed in natural killer T cells, γδ T cells, and mast cells. Based on ITK expression, it has been shown to have a predominant role in T cell function through T cell receptor signaling (TCR) [14,15].

Phosphorylated ITK activates several downstream targets, such as PLCγ1, leading to the formation of secondary messengers, such as IP_3_ and DAG, which further activate other signaling pathways, such as NF*k*B, NFATc1 and STAT3 [9,16,18]. These transcription factors translocate to the T cell nucleus and are involved in the differentiation of Th0 into Th17 cells. Th17 cells, after polarization, release several inflammatory cytokines, with IL-17A being the most predominant one [10,31]. To investigate the contribution of ITK in sepsis-mediated neuroinflammation, its expression in CD4+ T cells and the CNS was determined. Our data showed an increased expression of *p*-ITK and its downstream targets in CD4+ T cells and the CNS in mice with sepsis.

Immune cells in the systemic compartment are known to communicate with the CNS through the bidirectional trafficking of mediators. Peripheral immune cells have the potential to modulate the BBB and communicate with the CNS through various mechanisms following sepsis [7,19,32,33]. Sepsis can disturb the BBB, which may create an environment for the extravasation of leukocytes such as Th17 cells from the peripheral blood into the CNS. ITK signaling is required for the trafficking of Th17 cells across the BBB, which is a critical step in the induction of neuroinflammation [17]. Infiltrated Th17 cells may further activate resident CNS immune cells, such as microglia, causing them to produce a neuroinflammatory cocktail of mediators, such as cytokines, oxidants, proteases, and chemokines. These mediators further facilitate the influx of other immune cells into the brain, which aggravates the sepsis-linked neuroinflammatory state [23,34,35,36]. Consistently, Th17 cells and IL-17A protein levels along with other inflammatory mediators were increased in the periphery and the cerebral cortex of septic mice. BMS 509744-treated mice had a significant attenuation of IL-17A levels in the periphery and the CNS in sepsis survivor mice.

It is well-known that IL-17A can cause neuroinflammation through the activation of IL-17R, STAT3 and NF-kB [37,38]. IL-17A can cause the activation of resident brain immune cells such as microglial cells/astrocytes, which can promote the neuroinflammatory response through the production of pro-inflammatory cytokines, such as MCP-1, IL-6, and TNF-alpha from microglial cells, further worsening CNS dysfunction [39,40,41]. Overactivation of the microglia is involved in the progression of brain dysfunction, which could be responsible for a depression-like state and worse survival in septic patients. IL-17A has been shown to cause a depression-like state in animal models and human beings by causing neuroinflammation [35,40,41,42,43]. A recent study in rats has shown that Th17 cell recruitment into the CNS is required for the induction of a depression-like state [44]. The reduction in neuroinflammatory markers via a blockade of Th17/IL-17A signaling leads to cognitive improvements and anti-depressant effects in mice/rats [37,40,44]. Our study shows a reduction in IL-17A levels with a concurrent abatement of neuroinflammation by the ITK inhibitor, which may be responsible for the amelioration in sepsis-associated depression-like behavior.

Neutrophils are active participants in the innate immune response and are regarded as one of the major immune cells in sepsis-induced immune dysfunction [23]. Increased MPO activity (considered to be a marker of neutrophilic presence) in the cerebral cortex of sepsis survivor mice could be due to increased IL-17A levels. IL-17A has been shown to be a major chemoattract cytokine for neutrophilic transmigration across the BBB [35,43,45]. IL-17A-mediated neutrophil infiltration in the CNS may endanger neuronal cells by releasing oxidants through the activity of iNOS and MPO, thus promoting sepsis-associated CNS dysfunction [23,33,37]. Oxidative neuroinflammation was confirmed by the increased MPO activity, which is a hallmark of neutrophil infiltration in the brain. It has also been shown, earlier, during sepsis-induced neuroinflammation [24,43]. The ITK inhibitor mediated the downregulation of IL-17A, and subsequent oxidative stress in the CNS may protect neuronal cells from oxidative injury, which can partly mitigate sepsis-mediated behavioral problems.

ITK activity and Treg cell development have been shown to have a negative correlation. ITK plays a gatekeeper role in the development of Treg cells. Foxp3-expressing regulatory T cells (Treg) and Th17 cells share common requirements for their development such as the presence TGF-β [14,31]. Moreover, Th0 (naïve CD4+ T cells) deficient in ITK preferentially differentiate into Treg cells under the influence of Th17-differentiating cytokines in vitro [13,46]. Treg cells cause anti-inflammatory actions through multiple mechanisms such as the inhibition of DC maturation and the production of suppressive cytokines such as IL-10 [47]. A reduction in the Treg-related immune response in the CNS has been shown to cause a depression-like state in mice [39]. Our study showed elevated Foxp3/IL-10 levels and Treg cells in the CNS/periphery by the ITK inhibitor, which may counter the sepsis-mediated depression-like state in mice.

NF*k*B and Nrf2 signaling have been reported to have reciprocal relationships. Nrf2 is a ubiquitous transcription factor, which is heavily involved in the regulation of the antioxidant defense mechanism. Nrf2, by binding to its ARE in the nucleus, is responsible for the transcription of multiple antioxidant genes, such as hemoxygenase-1, superoxide dismutase-2, and thioredoxin [48]. Excessive activation of the NF*k*B pathway and ROS production can diminish Nrf2–ARE signaling in the nucleus leading to impaired antioxidant defense [41,49,50,51]. ITK inhibition caused the restoration of Nrf2 signaling in the cerebral cortex, which could lead to an amelioration of a depression-like state. Enhancing Nrf2 levels via genetic/pharmacological manipulation in mice has shown improved outcomes in sepsis [49,52].

This study has some limitations. Firstly, the CD4+ T cells were not isolated from the brain, which would have provided direct confirmation of ITK expression within these immune cells. Secondly, other types of T cells, which also express ITK as well as IL-17A, have not been analyzed in this study. Thirdly, sepsis-induced changes have been measured only in the cerebral cortex. Other brain areas, such as the hippocampus, amygdala, and hypothalamus, which may be affected by sepsis and play an important role in depression-like behavior, also need to be investigated in future studies. Moreover, the direct administration of the ITK inhibitor to the CNS through the intracerebroventricular route should also be investigated as it will show whether targeting ITK in CNS T cells has any effect on sepsis-induced depression-like behavior.

Sepsis in ICUs is one of the leading causes of deaths and morbidity throughout the world. The interaction between peripheral immune cells such as Th17 cells and the CNS during sepsis may cause multiple neurological outcomes. Sepsis survivors are linked to behavioral/mental derangements, such as depression, anxiety, delirium, loss of consciousness, and cognitive impairments in almost 70% of critically ill patients [5,7,36]. Our study shows for the first time that early intervention using the ITK inhibitor strategy may limit sepsis-induced neuroinflammation and depression via the regulation of the Th17/Treg balance, which may improve the long-term survival and better the prognosis of septic patients. 

## 4. Materials and Methods

### 4.1. Animals 

This investigation utilized male C57BL/6 mice following approval from the Animal Care and Research Committee of the College of Pharmacy, King Saud University. All experimental protocols were followed as per the recommended guidelines of the NIH for the utilization of animals. The mice used for the experiments were 48–50 weeks old (40–42 gm) and bred in-house at our college animal care facility. The mice were provided specific pathogen-free conditions with ventilated cages and a controlled environment (standard conditions of temperature and humidity with a 12 h light-dark cycle) for proper growth. The mice had access to food and water ad libitum. 

### 4.2. Induction of Sepsis and Drug Administration

To investigate the effect of ITK signaling on sepsis-induced neuroinflammation as well as depression-like symptoms, the aged mice were administered BMS 509744 (Tocris, Bristol, UK) at a dose of 10 mg/kg, i.p., at the time of the LPS injection. Thereafter, BMS 509744 was administered at the same dose once every day for 10 days. For the induction of sepsis, the mice were given an i.p. injection of LPS (derived from *E. coli*, O111:B4) at a dose of 2.5 mg/kg. There were four groups of mice, which were as follows: (1) Vehicle: mice treated with the vehicle (*n* = 6); (2) Vehicle + ITK-inhib (*n* = 6): mice treated with the vehicle and ITK inhibitor (10 mg/kg); (3) LPS (*n* = 8): mice administered at the set dose of 2.5 mg/kg as described above; and (4) LPS + ITK-inhib (*n* = 8): mice administered a dose of LPS (2.5 mg/kg) and treated with the ITK inhibitor (10 mg/kg). On days 8–10, the mice were utilized for different behavioral experiments followed by the sacrifice of the mice using a deep inhalational anesthesia of isoflurane. The organs (spleen/brain) were dissected out on day 10 for different molecular and biochemical assays as described below.

### 4.3. Tail Suspension Method 

The tail suspension method is one of the most-well known methods to assess depression-like behavior in mice, as described previously [53]. This test utilizes the paradigm of putting the animal in an unavoidable and uncomfortable situation via the tail suspension method. The mouse was suspended 25 cm above the ground by its tail using adhesive tape and the immobility time was recorded by a blinded observer for a period of 6 min. Immobility of the mouse was characterized by motionless hanging without active kicking and struggling. 

### 4.4. Sucrose Preference Test

The mice were provided access to two liquid bottles ad libitum. One bottle contained normal tap water and the other contained 2% sucrose in tap water. On the day of the experiment, the mice were provided with both water bottles for a period of 24 h. After that, the mice were returned to the home cage and the amount of water consumed from each bottle (sucrose/tap water bottle) was calculated. The mice were habituated to a consumption of 2% sucrose solution and normal tap water before the actual testing. The sucrose preference (%) was calculated as follows = (sucrose intake/total fluid intake) ×  100.

### 4.5. Marble Bury Behavior

The marble burying experiment was carried out to evaluate the depression-like behavior of mice [39]. In summary, each mouse was given a task to explore burying behavior for 30 min in a cage that had 20 marbles (placed in a pattern of 4 × 5 with a diameter of 15 mm for each marble) on top of 3–4 cm deep unperfumed bedding. A scientist blinded to the experimental groups counted the number of marbles that were buried by each mouse. If a marble was buried >50% in the bedding, then it was counted as a buried marble.

### 4.6. Measurement of Myeloperoxidase Activity in CNS

Myeloperoxidase (MPO) activity was measured in the cortex of different experimental groups as an indicator of neutrophilic inflammation, as described earlier [54,55]. Briefly, an MPO substrate buffer containing H_2_O_2_ (0.0005%) and O-dianisidine (0.167 mg/mL) in 50 mM potassium phosphate was incubated with the sample and allowed to stand for 20 min at 25 °C. The MPO activity was recorded by measuring the OD at 450 nm. The OD was normalized by the protein content of the respective sample.

### 4.7. Nrf2 Trans-Activation ELISA Assay in the CNS 

The Nrf2 trans-activation binding activity of the nuclear extracts of cortex was assessed utilizing a commercial Trans-AM Nrf2 kit (Active Motif, Carlsbad, CA, USA). Nuclear extract from the brain cortex of each mouse was prepared using a Nuclear Extract kit according to the protocol of the supplier (Active Motif, Carlsbad, CA, USA). In summary, this ELISA kit utilizes the coupling of Nrf2 present in the brain sample to its ARE, i.e., 5′-GTCACAGTACTCAGCAGAATCTG-3′. Nrf2/ARE binding activity in the nuclear extracts of cortex is directly proportional to the color development. The data were expressed in fold difference. 

### 4.8. Real-Time PCR

The cortex was isolated in a RNAlater^®^ (Thermo Fisher Scientific, Waltham, MA, USA) solution and used within a month after its isolation. The total RNA was isolated using the TRIzol method followed by checking its purity (OD:260/280 > 2) and concentration. The total RNA (1 µg) was then converted into cDNA via the reverse transcription method using a high-Capacity cDNA archive kit (Applied Biosystems, Waltham, MA, USA) as written before [45,54,55]. mRNA expression was conducted on the ABI PRISM 7500 sequence detection system (Applied Biosystems, Waltham, MA, USA) using the primers (GenScript, Piscataway, NJ, USA) for the following genes, i.e., iNOS, HO-1, Nrf2, IL-6, MCP-1, IL-17A, SOD2, and GAPDH (endogenous control). The relative mRNA expression in different brain samples was measured using the well-known ddCt method.

### 4.9. Preparation of Samples for Biochemical Measurements

The brain cortex from each mouse was homogenized in an ice-cold cell lysis buffer with a protease inhibitor cocktail (Thermo Fisher Scientific, Waltham, MA, USA) followed by centrifugation at 12,000 rpm for 20 min. The supernatant was then collected from each sample and used in the different assays mentioned below. Blood was collected via cardiac puncture in non-heparinized tubes after terminal anesthesia, and the serum was separated and stored at −80 °C for the IL-17A/IL-10 measurement.

### 4.10. Estimation of ITK, p-NFkB, and p-STAT3 Levels in the Cortex

Levels of the ITK and phospho-NF*k*B proteins in the cerebral cortex of different groups were measured using ITK (Aviva Systems Biology, San Diego, CA, USA), CST’s PathScan^®^ Phospho-NF*k*Bp65 (Ser536), and CST’s PathScan^®^ Phospho-STAT3 (Tyr705) (Cell Signaling Tech, Danvers, MA, USA) ELISA kits, respectively. ITK, *p*-NF*k*B, and *p*-STAT3 levels were measured in terms of the OD and later normalized with protein content with the respective samples. The results are expressed as the OD450/mg protein.

### 4.11. Estimation of Lipid Peroxides in Cortex

An assessment of the lipid peroxides in the cortex was performed as described before [56]. The data were expressed as nmol/mg protein. 

### 4.12. Estimation of Cytokines in Cortex/Serum

An assessment of IL-17A/IL-10 in the cortex/serum was carried out using commercially available ELISA kits (Biolegend, San Diego, CA, USA). The data were expressed as pg/mg protein (cortex) and pg/mL (serum). 

### 4.13. Flow Cytometry

The spleen was made into a single cell suspension for flow cytometry, as described earlier [45,55]. Briefly, the spleen was collected immediately after sacrifice and placed in a 70 μm stainless steel strainer, which was kept on top of a 50 mL conical tube containing RMPI-1640 medium. The spleen was then dilacerated with the piston of a syringe to collect a single cell suspension. Immune cells (1 million cells) in the splenic cell suspension were immunostained with monoclonal antibodies against cell surface receptors, i.e., CD4 coupled to APC-Cy7/FITC/APC (Biolegend, San Diego, CA, USA; Santa Cruz Biotech, Dallas, TX, USA). Immune cells were then permeabilized/fixed and processed for intracellular staining. Immune cells were then immunostained with monoclonal antibodies against intracellular proteins such as IL-10, *p*-ITK, *p*-NF*k*B, Foxp3, *p*-STAT3 and IL-17A coupled to APC/PE/FITC (Biolegend, San Diego, CA, USA; Cell Signaling Tech, Danvers, MA, USA). Immune cells were then acquired (10,000 events) on a flow cytometer and analyzed with Cytomics FC500 software (Beckman Coulter, Brea, CA, USA), as described earlier [45,55]. CD4+ T cells and their intracellular markers were analyzed in a lymphocytic gate on a forward and side scatter plot.

### 4.14. Statistical Analysis

The data were shown as the Mean ± SE and the significance level was set at *p* < 0.05 for statistical analyses. Molecular/biochemical data were analyzed with one-way ANOVA followed by Tukey’s post-hoc test for multiple comparisons. Brown–Forsythe is used automatically by GraphPad Prism 9 software for testing the homogeneity of variances; this test showed *p >* 0.05 for the analyzed parameters. All statistical calculations were analyzed using GraphPad Prism 9 (GraphPad Software, San Diego, CA, USA).

## 5. Conclusions

Sepsis in ICUs is one of leading causes of deaths and morbidity throughout the world. The interaction between peripheral immune cells such Th17 cells and CNS during sepsis may cause multiple neurological outcomes. Sepsis survivors are linked to behavioral/mental derangements such as depression, anxiety, delirium, loss of consciousness, and cognitive impairments in almost 70% of critically ill patients [5,7,30]. Our study shows for the first time that early intervention by ITK inhibitor strategy may limit sepsis-induced neuroinflammation and depression by regulation of Th17/Treg balance which may improve the long-term survival and better the prognosis of septic patients. 

## Data Availability

All data presented in this study are available on reasonable request from the corresponding author.
